# Activity of Gallium Meso- and Protoporphyrin IX against Biofilms of Multidrug-Resistant *Acinetobacter baumannii* Isolates

**DOI:** 10.3390/ph9010016

**Published:** 2016-03-17

**Authors:** David Chang, Rebecca A. Garcia, Kevin S. Akers, Katrin Mende, Clinton K. Murray, Joseph C. Wenke, Carlos J. Sanchez

**Affiliations:** 1 Infectious Disease Service, Department of Medicine, San Antonio Military Medical Center, Ft. Sam Houston, San Antonio, TX 78234, USA; kevin.s.akers.mil@mail.mil (K.S.A.); katrin.mende.ctr@mail.mil (K.M.); clinton.k.murray.mil@mail.mil (C.K.M.); 2 United States Army Institute of Surgical Research, Extremity Trauma and Regenerative Medicine, Ft. Sam Houston, San Antonio, TX 78234, USA; rebecca.a.garcia28.ctr@mail.mil (R.A.G.); joseph.c.wenke.civ@mail.mil (J.C.W.); sanchezcj@livemail.uthscsa.edu (C.J.S.J.); 3 Infectious Disease Clinical Research Program, Uniformed Services University of the Health Sciences, Bethesda, MD 20814, USA

**Keywords:** *Acinetobacter baumannii*, biofilm, multidrug-resistant, gallium mesoporphyrin IX, gallium protoporphyrin IX

## Abstract

*Acinetobacter baumannii* is a challenging pathogen due to antimicrobial resistance and biofilm development. The role of iron in bacterial physiology has prompted the evaluation of iron-modulation as an antimicrobial strategy. The non-reducible iron analog gallium(III) nitrate, Ga(NO_3_)_3_, has been shown to inhibit *A. baumannii* planktonic growth; however, utilization of heme-iron by clinical isolates has been associated with development of tolerance. These observations prompted the evaluation of iron-heme sources on planktonic and biofilm growth, as well as antimicrobial activities of gallium meso- and protoporphyrin IX (Ga-MPIX and Ga-PPIX), metal heme derivatives against planktonic and biofilm bacteria of multidrug-resistant (MDR) clinical isolates of *A. baumannii in vitro*. Ga(NO_3_)_3_ was moderately effective at reducing planktonic bacteria (64 to 128 µM) with little activity against biofilms (≥512 µM). In contrast, Ga-MPIX and Ga-PPIX were highly active against planktonic bacteria (0.25 to 8 µM). Cytotoxic effects in human fibroblasts were observed following exposure to concentrations exceeding 128 µM of Ga-MPIX and Ga-PPIX. We observed that the gallium metal heme conjugates were more active against planktonic and biofilm bacteria, possibly due to utilization of heme-iron as demonstrated by the enhanced effects on bacterial growth and biofilm formation.

## 1. Introduction

*Acinetobacter baumannii* is an important and problematic pathogen, emerging as a significant cause of community-acquired and nosocomial infections, particularly amongst critically ill patients in intensive care units [1,2,3]. *A. baumannii* has also been recognized as a major cause of combat-related infections accounting for up to 36% of infections resulting from injuries sustained by military service personnel in the Middle East [4,5,6]. Notably, infections due to *A. baumannii* have become increasingly difficult to treat given the rapid emergence of antimicrobial resistance in clinical isolates [7,8,9], necessitating the use of new antibiotic treatment strategies and/or older antimicrobial agents retaining activity against the majority of isolates. These agents include colistin and minocycline [10,11]. Importantly, there are already reports of infections caused by multidrug-resistant (MDR) *A. baumannii* displaying resistance to the few currently used antimicrobials for treatment [12,13].

In addition to the rapid acquisition of antimicrobial resistance, the ability of clinical isolates of *A. baumannii* to form biofilms has also been recognized as a significant factor contributing to the success of this pathogen [1,14,15]. Biofilms are groups of bacteria associated with a surface (both abiotic and biotic) and embedded within a self-produced polymeric extracellular matrix comprised of a mixture of proteins, polysaccharides and extracellular DNA [16]. As a direct and clinically important consequence of the biofilm mode of growth, bacteria within biofilms often develop an intrinsic resistance to conventional antibiotics, typically 100–1000-fold compared to their planktonic counterparts, thus limiting the effectiveness of conventional antibiotics [17,18]. For *A. baumannii*, biofilm formation has been implicated as an important mechanism enhancing virulence and has been associated with the development of chronic wound infections [1,15]. Furthermore, colonization and persistence of *A. baumannii* within biofilms has been linked to the development of antimicrobial resistance amongst strains [19,20] and has been suggested to be a significant reservoir of organisms contributing to outbreaks in both civilian and military populations [21,22]. The lack of effective drugs for the treatment of infections caused by drug resistant *A. baumannii* and the ability to form biofilms highlights the need for the development of novel therapeutic options.

A critical characteristic shared by multiple pathogens is the acquisition of ferric iron from the host where bioavailability is normally kept extremely low (<10^−18^ M) [23]. In response to the low availability of iron, *A. baumannii* has evolved numerous strategies enabling iron acquisition. These include the expression of high affinity siderophores such as acinetobactin and fimsbactin as well as ferrous and/or heme uptake systems used to overcome the iron restrictions within the host [24,25,26]. Given the central role of ferric iron to bacterial growth, virulence, and biofilm formation, the modulation of iron metabolism could potentially be used as an antimicrobial therapy for *A. baumannii*. While several studies have demonstrated the success of modulating iron availability for various pathogens using iron chelators, including desferoxamine (DFO), deferiprone, and 2,2-dipyridyl (DIP) as an antimicrobial strategy, only recently has this approach been extended to *A. baumannii.* Reports have demonstrated their limited and highly variable antimicrobial activity against clinical strains [27,28]. In contrast, the use of the non-reducible ferric iron analog, gallium (III) has been shown to be active against Gram-negative and Gram-positive bacteria *in vitro* under iron limited conditions. It is hypothesized that gallium (III) has the ability to efficiently compete with iron (III) for binding and to interfere with bacterial metabolism [29,30]. Gallium nitrate (Ga(NO_3_)_3_), the active component of the FDA-approved drug Ganite used for the treatment of bone degenerative diseases [31], has been reported to have antimicrobial activity against *A. baumannii* inhibiting bacterial growth *in vitro* as well as reducing bacterial burden and associated lethality *in vivo* [27,32]. Additionally, there have been *in vitro* studies demonstrating antibacterial effects among different porphyrins, and, in particular, gallium mesoporphyrin and protoporphyrin demonstrated against *Neisseria gonorrhoeae* [33,34]*.* While studies have supported the use of Ga(NO_3_)_3_ as a potential antimicrobial therapy for *A. baumannii*, more recent studies have shown that utilization of heme-iron sources by clinical strains of *A. baumannii* can contribute to the development of tolerance to gallium alone [35]. Given these observations, as well paucity of data on activity of gallium on biofilms, herein we evaluated the antimicrobial activities of the gallium heme-porphyrins, gallium mesoporphyrin IX (Ga-MPIX) and gallium protoporphyrin IX (Ga-PPIX), against planktonic and biofilm bacteria of clinical MDR *A. baumannii* isolates.

## 2. Results

### 2.1. Iron Sources Differentially Affect Bacterial Growth and Biofilm Formation of A. Baumannii In Vitro

**Figure 1:**
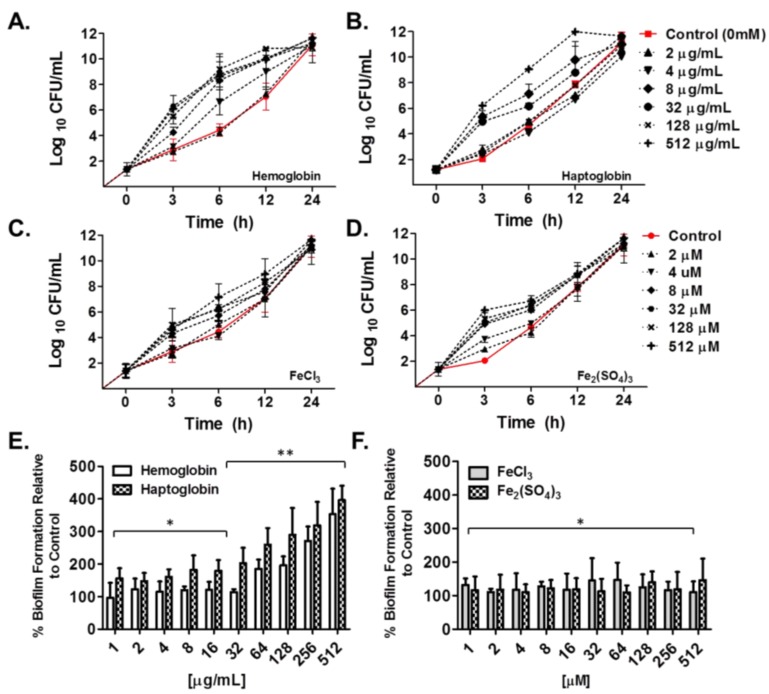
Effect of heme iron and non-heme iron sources on bacterial growth and biofilm formation *in vitro*. *A. baumannii* strain 17978 was grown for up to 24 h in MHB II (0.3 g/L) supplemented with increasing concentrations of (**A**) human hemoglobin; (**B**) haptoglobin (0–512 µg/mL) or (**C**) FeCl_3_, or (**D**) Fe_2_(SO_4_)_3_ (0 to 50 μM). Growth over time was expressed as the mean log value ± SD. Percentage of biofilm formation of *A. baumannii* strain 17978, as determined by the crystal violet method, relative to untreated controls, in the presence of heme iron (**E**) and non-heme iron sources (**F**) after 24 h of growth in MHB II (0.3 g/L) under static conditions. Values are expressed as the mean ± SD. Experimental assays are representative of at least three independent experiments. * *p* < 0.001 and ** *p* < 0.00001 relative to the mean value of biofilm formation for strains grown under non-supplemented conditions.

**Figure 2:**
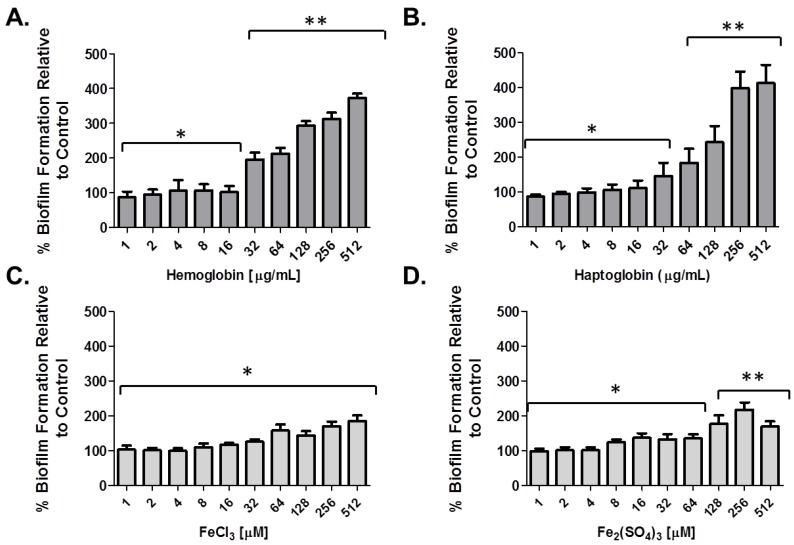
Differential effects of iron sources on biofilm formation of clinical isolates of *A. baumannii*. Mean increases of biofilm formation of clinical isolates of *A. baumannii* (*n* = 12), as determined by the crystal violet method, relative to untreated controls, in the presence of heme iron (1–512 µg/mL; (**A)** and (**B**) and non-heme iron sources (1–512 µM; **C** and **D**) after 24 h. Values are representative of the mean ± SD, of relative % increase of biofilm formation of each of the individual strains to untreated control groups. Experimental assays are representative of at least three independent experiments. * *p* < 0.001 and ** *p* < 0.00001 relative to the mean value of biofilm formation for strains grown under non-supplemented conditions.

**Figure A1:**
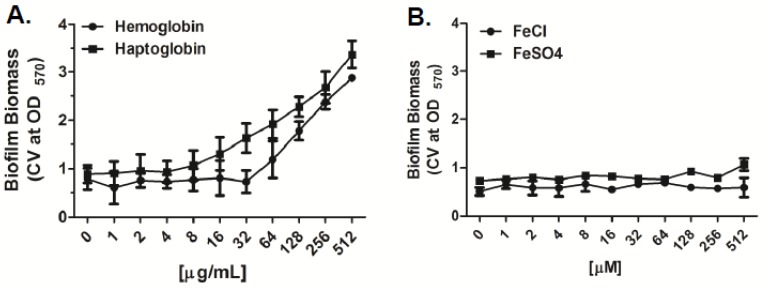
Effect of heme iron and non-heme iron sources on biofilm formation *in vitro.* *A. baumannii* strain 17978, was grown for up to 24 h in MHB II (0.3 g/L) supplemented with increasing concentrations of (**A**) human hemoglobin or haptoglobin (0–512 µg/mL) or (**B**) FeCl_3_, or Fe_2_(SO_4_)_2_ (0 to 50 μM). Biofilm biomass, is reported as the measured absorbance of solubilized crystal violet of stained biofilms at 570 nm (CV at OD_570_), Values represent the mean ± SEM of three independent experiments.

Planktonic as well as biofilm growth can be modulated by numerous factors including carbon sources, temperature, as well as iron availability [23,36]. Given the recent observations of utilization of heme iron by clinical isolates of *A. baumannii* and its contribution to Ga(NO_3_)_3_ tolerance *in vitro* [35], we initially evaluated the effect of heme iron and non-heme iron sources on bacterial growth as well as biofilm formation *in vitro*. Exposure of *A. baumannii* strain 17978 to human hemoglobin (Hgb) and haptoglobin (Hp) was observed to drastically enhance bacterial growth in a concentration dependent manner compared to bacteria grown under non-supplemented conditions (Figure 1A,B). Specifically supplementation with Hgb and Hp at concentrations ≥ 8 µg/mL and ≥ 32 µg/mL, respectively, supported rapid bacterial growth during the first 6 h reaching population density limits by 12 h. In contrast, supplementation of media with the iron salts, FeCl_3_ and Fe_2_(SO_4_)_3_, both examples of non-heme iron sources, were observed to have only moderate effects on bacterial growth, compared to bacteria grown under normal conditions, even with supplementation of media as high as 512 µM (Figure 1C,D). In line with these results, biofilm formation of *A. baumannii* 17978 relative to biofilms grown under un-supplemented conditions, were also drastically enhanced in the presence of Hgb and Hp, compared to supplementation with the non-heme iron sources, although these agents also enhanced biofilm formation relative to untreated control groups (Figure 1E,F; Figure A1). Supplementation of media with Hgb and Hp enhanced biofilm formation in a concentration dependent manner between 300% and 500% compared to biofilms grown under non-supplemented conditions (Figure 1E), whereas supplementation of media with the iron salts at concentrations up to 512 µM, was observed to enhance biofilm formation to a much lesser extent—up to 200% (Figure 1F). Importantly, the effect of heme iron on biofilm formation was strain independent as supplementation with Hgb and Hp was also observed to have a similar impact on biofilm formation of the collection of MDR isolates tested herein (*n* = 12), compared to the iron salts (Figure 2).

### 2.2. Activity of Gallium Compounds on Planktonic Bacteria

**Figure 3:**
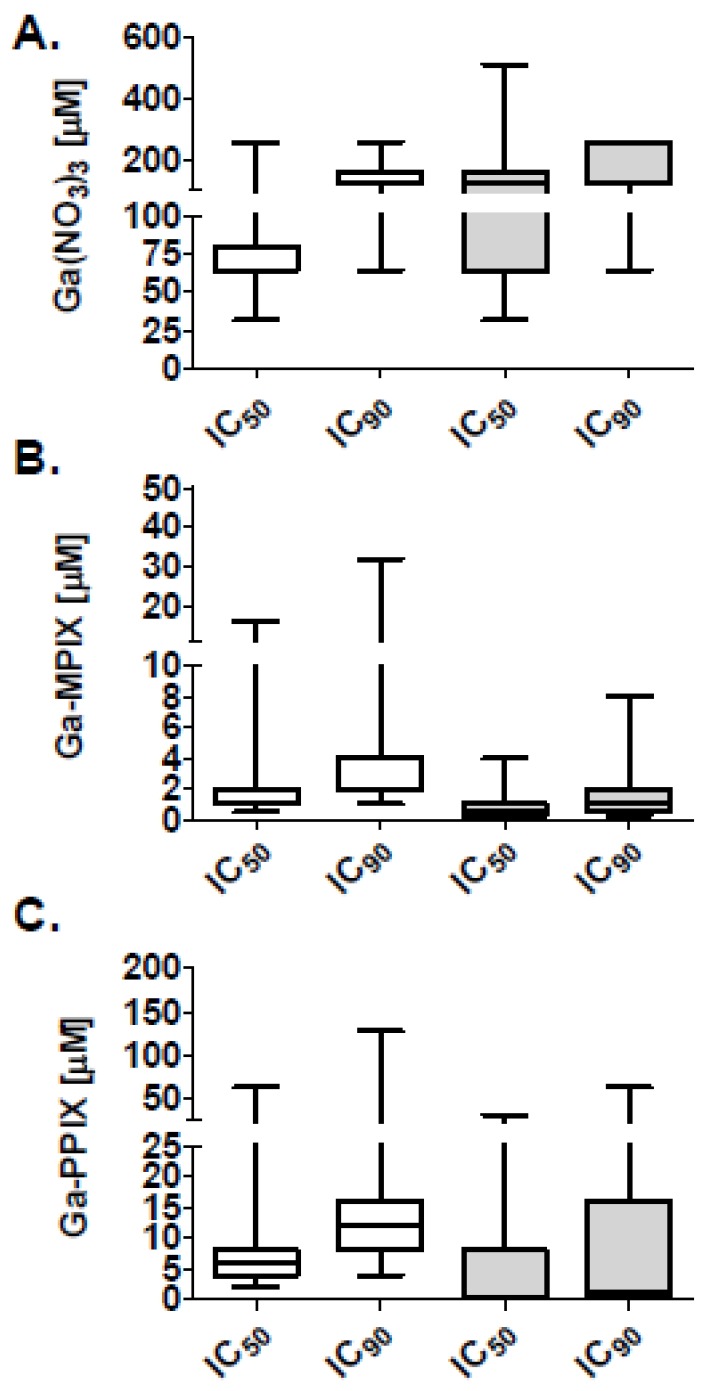
Inhibitory activities of Ga-MPIX and Ga-PPIX on a planktonic MDR *A. baumannii* isolates. Genotypically distinct isolates of *A. baumannii* (*n* = 13) were grown in the iron deplete medias, MHB II (0.3 g/L) (un-shaded boxes) or RPMI1640 (shaded boxes), and exposed to different concentrations (0–512 µM) of (**A**) Ga(NO_3_)_3_; (**B**) Ga-MPIX; or (**C**) Ga-PPIX for 24 h. Plots show the concentrations (μM) required to reduce viable bacteria of individual isolates by 50% (IC_50_) and 90% (IC_90_). Boxes represent medians; whiskers represent range of strains tested.

Having observed the difference in effect of heme *versus* the non-heme iron sources on planktonic growth we next evaluated the effect of the gallium (III) heme porphyrins, Ga-MPIX and Ga-PPIX, as well as Ga(NO_3_)_3_, a non-heme iron derivative of gallium (III) on planktonic growth for all clinical strains of *A. baumannii* by performing broth microdilution assays in the iron deplete medias, including MHB II (0.3 g/L) and Roswell Park Memorial institute Media (RPMI) and determined the IC_50_ and IC_90_ after 24 h of growth (Figure 3). The median IC_50_ and IC_90_ for Ga(NO_3_)_3_ were 64 and 128 µM, respectively, in MHB II (0.3 g/L), and 128 and 256 µM, respectively, in RPMI. In contrast, median IC_50_ and IC_90_ for Ga-MPIX were 1 and 2 µM, respectively, in MHB II (0.3 g/L) and 0.5 and 1µM, respectively, in RPMI. For Ga-PPIX, median IC_50_ and IC_90_ were 8 and 16 µM, respectively, in MHB II (0.3 g/L) and 2 and 4 µM, respectively, in RPMI.

### 2.3. Antibiofilm Activity of Gallium Compounds

**Figure 4:**
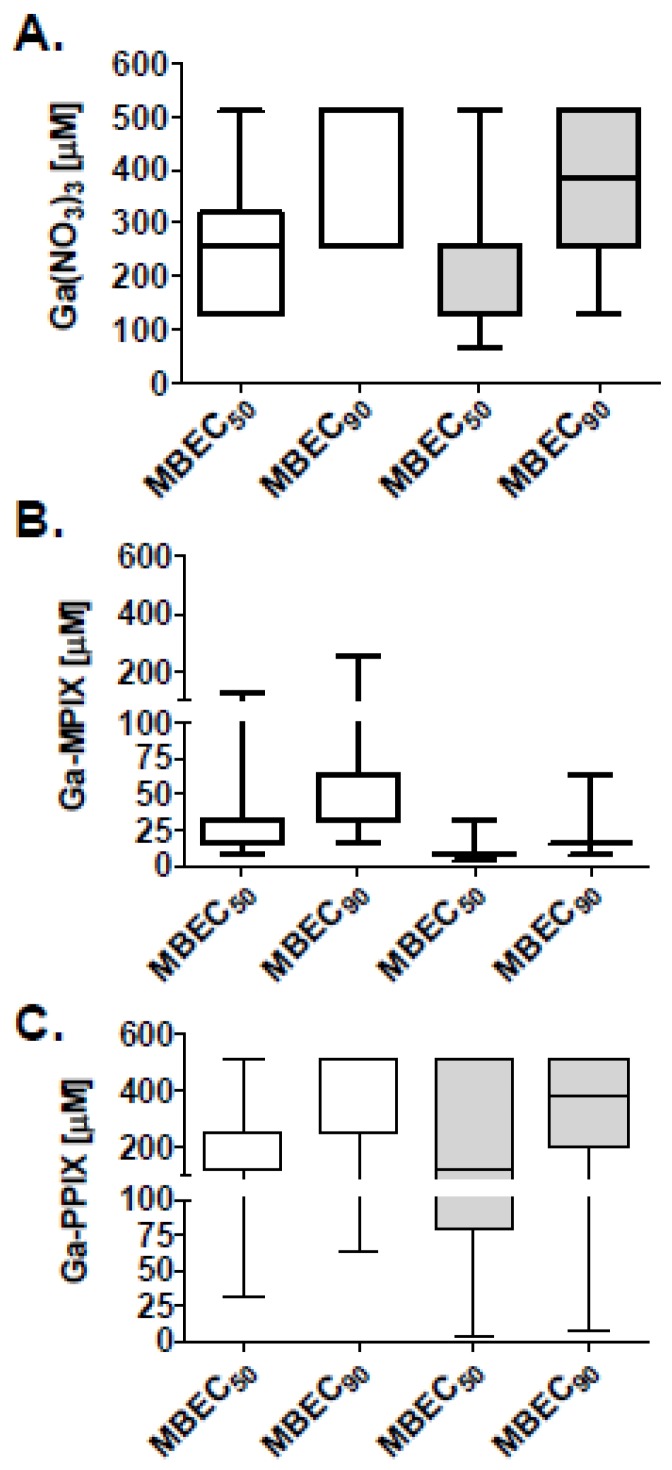
Activity of Ga-MPIX and Ga-PPIX against biofilms of MDR *A. baumannii* isolates. Biofilms of isolates of *A. baumannii* (*n* = 13) were grown on individual pegs of an MBEC device for 24 h followed by exposure to different concentrations (0–512 µM) of (**A**) Ga(NO_3_)_3_; (**B**) Ga-MPIX; or (**C**) Ga-PPIX for 24 h in the iron deplete medias, MHB II (0.3 g/L) (un-shaded boxes) or RPMI1640 (shaded boxes). Plots show the concentrations (μM) required to reduce viable bacteria within biofilms of individual isolates by 50% (MBEC_50_) and 90% (MBEC_90_) to untreated controls. Boxes represent medians; whiskers represent range of strains tested.

To determine whether the gallium heme porphyrins were also effective against biofilm bacteria, we evaluated the activity against bacteria within established biofilms utilizing the Minimum Biofilm Eradication Concentration (MBEC) model, which is an established model for susceptibility testing of biofilms [37], and determined the MBEC_50_ and MBEC_90_ (Figure 4). In contrast to the activity against planktonic bacteria, the overall concentrations required to effectively reduce viable bacteria within biofilms to 50% and 90% of untreated controls was much higher for all of three gallium compounds. Median MBEC_50_ and MBEC_90_ for Ga(NO_3_)_3_ were 256 and >512 µM, respectively, in MHB II (0.3 g/L), and 128 and 256 µM, respectively, in RPMI. In contrast, the median MBEC_50_ and MBEC_90_ for Ga-MPIX were 16 and 32 µM, respectively, in MHB II (0.3 g/L), and 8 and 16 µM, respectively, in RPMI, whereas, for Ga-PPIX, median MBEC_50_ and MBEC_90_ were 128 and 256 µM, respectively, in MHB II (0.3 g/L), and 128 and 256 µM, respectively, in RPMI.

### 2.4. Assessment of the Cytotoxicity of Gallium Compounds

**Figure 5:**
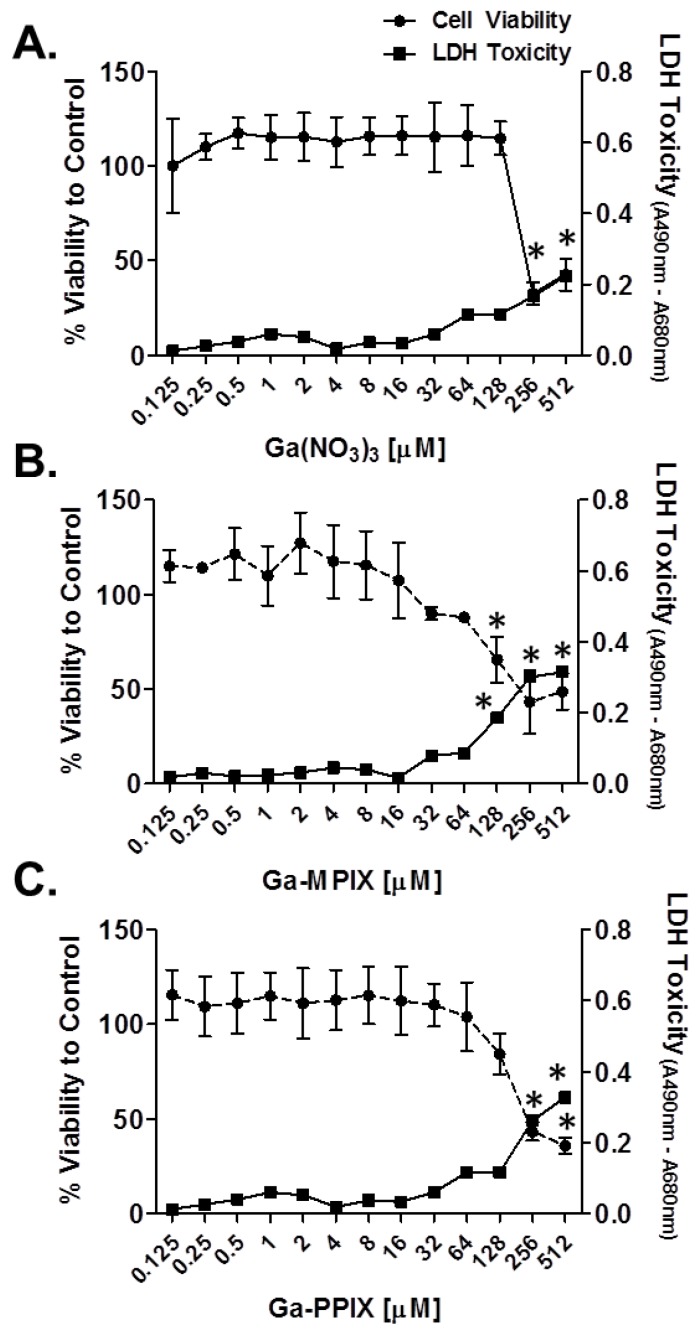
Evaluation of the toxicity of gallium porphyrins on primary human fibroblasts. Human fibroblasts were exposed to different concentrations (0–512 µM) of (**A**) Ga(NO_3_)_3_; (**B**) Ga-MPIX; or (**C**) Ga-PPIX for 24 h. Cell viability was measured using the cell titer flour assay and viability was expressed as a percentage compared to untreated control groups. Cytotoxicity was evaluated by measuring the amount of lactate dehydrogenase (LDH) in culture supernatants of cells (*A*_490nm_) treated with the gallium compounds. Values are expressed as the mean ± SD of three independent experiments. * *p* < 0.01 relative to the untreated control group.

To assess the biocompatibility of the different gallium compounds, we evaluated cytotoxicity and viability of human dermal fibroblasts exposed overnight to increasing concentrations of the gallium compounds. Individual compounds were observed to have differential effects on cell viability and toxicity on fibroblasts, which was largely concentration dependent (Figure 5). Of the three compounds tested, Ga(NO_3_)_3_ had the least negative effect on cells, with concentrations up to 128 µM being well tolerated and having only moderate, albeit insignificant, losses in cell viability and low levels of extracellular LDH detected within supernatants compared to the untreated control groups (Figure 5A). Exposure of cells to Ga-MPIX was tolerated up to concentrations of 64 µM with >80% cell viability and only moderate increases in LDH levels detected after 24 h, whereas exposure of cells to concentrations of ≥128 µM was associated with significant loses in cell viability, >60%, and significant increases in detected LDH levels compared to untreated control groups (Figure 5B). Similar to the effect of Ga-MPIX, exposure of cells to Ga-PPIX at concentrations ≤128 µM were compatible with cells, whereas exposure to concentrations ≥256 µM was associated with ≥50% loss of cell viability and increased levels of detected LDH levels (Figure 5C).

## 3. Discussion

*Acinetobacter baumannii* has emerged as a significant cause of nosocomial infections amongst the critically ill; moreover, it has been recently identified as a leading cause of combat-related bacterial wound infections [1,2,3,5,14]. Lately, the clinical management of *A. baumannii* infections has become challenging in part due to the ability of the organism to rapidly acquire antimicrobial resistance as well as the capacity to develop and persist within biofilms, which has been suggested as a mechanism in the development of antimicrobial resistance [19,20]. Recently, the use of the non-reducible iron analog gallium (III) in the form of the salt Ga(NO_3_)_3_, the FDA approved drug used to treat hypercalcemia of malignancy, Paget’s disease of bone, parathyroid carcinoma and osteolytic bone metastases [31], has been shown to have antimicrobial activity *in vitro* against many clinically significant bacteria, including *A. baumannii* [27,32]. Though Ga(NO_3_)_3_ therapy has been suggested to be potentially used for the treatment of *A. baumannii* infections, recent studies demonstrating the development of tolerance to gallium by clinical isolates due to utilization of heme iron have indicated that there may be direct limitations to its application [35]. In light of these observations, herein we evaluated the antimicrobial activities of two recently described gallium porphyrin conjugates, Ga-MPIX and Ga-PPIX, against planktonic and biofilm bacteria of MDR clinical strains of *A. baumannii in vitro*, and assessed the potential toxicity associated with these agents using human cell lines *in vitro*.

While there has been limited success with the use of iron chelators directly as an antimicrobial therapy [28], the use of the iron analog gallium (III), more commonly as the salt form Ga(NO_3_)_3_, has been shown to have antimicrobial activity against MDR isolates of *A. baumannii in vitro* [27,32]. In these studies the antimicrobial activity of Ga(NO_3_)_3_ was observed to be strain and concentration dependent, with significant activity reported between 2 and 100 µM. While antimicrobial activity was observed against the MDR isolates exposed to Ga(NO_3_)_3_ in our study, much higher concentrations, between 64 and 256 µM, were required. The discrepancies between our findings and those previously published, could have been due to differences in the strains used as well as the media choice for susceptibility testing. However, antimicrobial activity of Ga(NO_3_)_3_, was observed to be similar in the two different culture medias evaluated, excluding the possibility that these differences were primarily due to the media choice. As strains have been shown to utilize different sources of iron which can influence susceptibility to Ga(NO_3_)_3_ [35], the higher concentrations may reflect differences of the individual strains tested herein to utilize these iron sources. Notably, the variability in activity between isolates and studies highlight the potential limitations for use of Ga(NO_3_)_3_ against *A. baumannii* isolates. Importantly, and in contrast to activity against planktonic bacteria, Ga(NO_3_)_3_ had little to no antimicrobial activity against biofilm bacteria requiring concentrations often exceeding the test ranges evaluated herein. While the activity of Ga(NO_3_)_3_ against biofilms of *A. baumannii* has not been previously described, our findings are in line with studies evaluating antibiofilm activity of Ga(NO_3_)_3_ in *Pseudomonas aeruginosa* where concentrations ~10-fold higher (1 mM) than those observed for bacterial growth inhibition (100 µM), were required for activity against biofilms [38]. As the recommended dosing regimens for intravenous Ga(NO_3_)_3_ treatment of cancer-related hypercalcemia ensure peak serum concentrations of ~28 µM [39,40]**,** our findings indicate that concentrations exceeding this may have to be considered to ensure antimicrobial activity, which could limit direct applications given associated reports of systemic toxicity at concentrations >200 µM [39].

In contrast to Ga(NO_3_)_3_, the gallium porphyrin conjugates, Ga-MPIX and Ga-PPIX, were observed to have better antimicrobial activity against planktonic isolates of *A. baumannii*, with concentrations between 0.5 to 8 µM and 2 to 64 µM, respectively, reducing viable bacteria to near the limits of detection. Interestingly, although both gallium porphyrins showed activity against planktonic bacteria, only Ga-MPIX was observed to have antimicrobial activity against biofilm bacteria at concentrations only three times those required for inhibition of planktonic bacteria (8 µM compared to 32 µM, respectively), whereas Ga-PPIX had little to no activity against biofilms similar to what was observed for Ga(NO_3_)_3_. Consistent with these observations, previous studies examining the effects of gallium porphyrin conjugates to those of Ga(NO_3_)_3_ have reported similar findings. In a study by Stojiljkovic *et al.*, Ga-PPIX at concentrations 100-fold lower than gallium alone, had broad antimicrobial activity against Gram-negative and Gram-positive pathogens, including *Yersinia enterocolitica*, methicillin-resistant *Staphylococcus aureus* and *Mycobacterium smegmatis* [41]. Similarly, both Ga-PPIX and Ga-MPIX have been recently reported to inhibit growth at concentrations ~three-fold less than gallium alone (<10 µM), against isolates of *Staphylococcus epidermidis* and *P. aeruginosa* [42]. In the same study, Ga-MPIX and Ga-PPIX were also observed to inhibit bacterial attachment and prevent biofilm formation at concentrations required for planktonic inhibition. Although activity against established biofilms was not directly evaluated in this study, our findings are consistent with these and demonstrate that gallium porphyrin conjugates retain activity against biofilm bacteria. While the differences in the activity against biofilms between the gallium porphyrins cannot be directly explained, issues relating to solubility of Ga-PPIX may have been a contributing factor. Additionally, it is likely that the structural differences in the side chains of protopophyrin and mesopophyrin also contribute to increased biofilm activity, as this has been demonstrated with structural modifications to the porphyrins resulting in increased MIC values [33]. Importantly, and in contrast to Ga(NO_3_)_3_, the consistency in activity against clinical pathogens other than *A. baumannii*, as well as low concentrations required for antimicrobial activity suggest that the gallium porphyrins may be advantageous to Ga(NO_3_)_3_ for use against MDR *A. baumannii*. Importantly, recent studies have demonstrated that Ga-PPIX susceptibility to clinical strains of *A. baumannii* is independent of free-iron content of the media, indicating applications of protoporphyrins under conditions where iron-restricted conditions may not be ideal, such as in wounds [43]. Importantly, while activity was observed following exposure of the *A. baumannii* biofilms to the gallium porphyrins complexes, it is important to note the limitations of our findings, specifically that our major outcomes evaluated the remaining bacteria attached to pegs and did not directly give indications as to whether these differences were due to a dispersal or bactericidal effect. Future studies focused on the mechanisms behind the antimicrobial activity are warranted.

In response to low iron availability within the host, *A. baumannii* employs a number of mechanisms for capturing iron and/or iron-protein complexes, including the expression of siderophores as well as ferrous iron and heme uptake systems [25,26]. While several of these systems act simultaneously, it is not unusual for strains of *A. baumannii* to efficiently utilize various iron sources over others given the plasticity of the bacterial genome amongst strains [44,45]. In a recent study evaluating a highly virulent strain of *A. baumannii*, LAC-4, it was observed that the ability of this strain to utilize heme iron much more efficiently compared to other strains allowed for its rapid growth in serum and contributed to the enhanced virulence observed *in vivo* [35]. Interestingly, LAC-4 also exhibited a high tolerance to Ga(NO_3_)_3_ but was highly susceptible to Ga-PPIX, which was, in part, due to the preference for heme iron utilization. Our results demonstrating the enhanced activity of the gallium porphyrins against the clinical isolates may, in part, be explained by similar mechanisms as our initial analysis demonstrated the drastic effect of heme iron sources on both bacterial growth and biofilm formation. It should be mentioned that the exact mechanism of the porphyrins is still unknown and the heme irons used in this study (hemoglobin and haptoglobin) are complexed with heme; thus, a direct comparison between the heme irons and the porphyrins cannot be directly made. Although we cannot directly exclude the activity of other iron acquisition mechanisms, our observations indicate that utilization of heme iron sources may be a shared characteristic amongst clinical isolates, albeit to varying degrees and even dependent on phenotype, given the differences in susceptibilities of the individual strains.

While Ga(NO_3_)_3_ has been extensively evaluated and determined to have minimal toxicity [40,46], much less is known about the toxicity of Ga-MPIX and Ga-PPIX. In a study by Stojiljkovic *et al.*, evaluation of the toxicity of Ga-PPIX up to 150 µg/mL (~150 µM) was not observed to be toxic to human fibroblasts, kidney (MDCK cells), and/or intestinal (CaCO-2) cell lines. Furthermore, in Balb/c mice, intraperitoneal injections into mice (25–30 mg/kg) followed by additional doses of 10–12 mg/kg were also not observed to affect the health and/or behavior of mice [41]. Similar to these findings, we observed that only exposure of fibroblasts to concentrations ≥128 µM of Ga-MPIX and Ga-PPIX were associated with significant toxicity. This is in-line with recent studies showing toxicity of Ga-PPIX only when exposed to concentrations 16-fold higher than those demonstrating antibacterial properties [43]. While toxicity was detected *in vitro*, previous studies evaluating the compatibility of Ga-PPIX *in vivo* indicate that these agents may be well tolerated. Future studies utilizing *in vivo* models are necessary to further characterize the compatibility and potential use of these agents for clinical settings.

## 4. Experimental Section

### 4.1. Reagents

Gallium (III) nitrate (Ga(NO_3_)_3_), iron (III) chloride (FeCl_3_), iron (III) sulfate (Fe_2_(SO_4_)_3_), human hemoglobin (Hgb) and human haptoglobin (Hp), hemoglobin stabilized, were purchased from Sigma-Aldrich (St. Louis, MO, USA). Gallium (III) mesoporphyrin IX (Ga-MPIX) and gallium (III) protoporphyrin IX (Ga-PPIX) were purchased from Frontier Scientific (Logan, UT, USA). All reagents were prepared for use in the experimental assays according to the recommendations of the manufacturer.

### 4.2. Bacterial Strains and Culture Conditions

**Figure A2:**
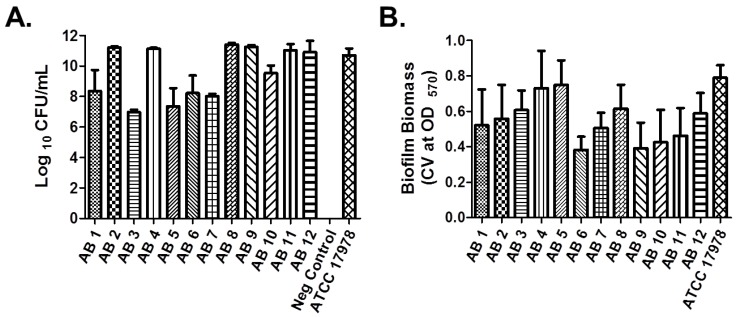
Screening of biofilm formation amongst clinical isolates of *A. baumannii* used in this study. (**A**) *A. baumannii* isolates were grown in MHB II broth at 37 °C and then incubated in the MBEC device overnight as previously described in the methods used in 4.5. Results shown are colony counts recovered from individual pegs after sonication of biofilms on MBEC plates and expressed as Log_10_; (**B**) screening of biofilm formation amongst the clinical isolates assessed by quantitating biofilm biomass by measuring the absorbance of ethanol solubilized crystal violet at 570 nm (CV at OD_570_) of previously stained biofilms. Bars represent the mean ± SEM of three independent experiments.

A convenience sample of twelve genetically distinct clinical isolates of *A. baumannii* were prospectively acquired from a strain collection isolated from wound infections of the upper and lower extremities of injured U.S. military personnel as part of the Trauma Infectious Disease Outcomes Study and unrelated to research (Table 1) [47]. A commercially available strain of *A. baumannii*, ATCC 17978, was also used as in this study. All strains, including the reference strain, was independently assessed for biofilm formation (Appendix Figure A2). Organisms were defined as MDR if they exhibited resistance to at least three of the major antibiotic classes (including aminoglycosides, β-lactams, carbapenems, and fluoroquinolones). Antimicrobial susceptibility testing was performed using the BD Phoenix™ system (Becton-Dickinson, Franklin Lakes, NJ, USA) per the manufacturer’s instructions. Resistance and susceptibilities to various drugs tested are reflective of the clinical breakpoints set by the Clinical and Laboratory Standards Institute (CLSI) as described by the performance standards for antimicrobial susceptibility testing (M100-S25, 2015). Bacterial isolates were recovered from frozen storage at −80 °C and sub-cultured on blood agar plates (Remel, Lenexa, KS, USA.) overnight at 37 °C prior to each experimental assay. Bacteria cultures were grown in cation adjusted Mueller-Hinton Broth (MHB II) with agitation at 37 °C. All isolates utilized in this study were assessed and determined to be phenotypically positive for biofilm formation (Appendix Figure A2).

### 4.3. Effects of Iron Sources on Bacterial Growth and Biofilm Formation

Effects of iron sources on bacterial growth were performed by evaluating bacterial growth over time in the presence of various heme and non-heme iron sources covering ranges previously observed to effect bacterial growth *in vitro* [49,50]. For the growth experiments, bacteria were grown as described above, 10^3^ cells were added to MHB II containing increasing concentrations of Hgb, Hp, FeCl_3_, or Fe_2_(SO_4_)_3_, and incubated at 37 °C for up to 24 h. At various time points after inoculation (0, 3, 6, 12, 24 h), 100 µL of bacterial suspensions were collected, serially diluted, and plated onto MHB agar to enumerate the number of viable bacteria. The effect of iron sources on biofilm formation was also examined using the static, semi-quantitative 96-well plate model as previously described [51,52]. Briefly, bacteria were grown in MHB II to an optical density of 0.1 at 600 nm (~10^8^ CFU/mL), diluted 1:100 into media in the presence of heme and non-heme iron sources at increasing concentrations as described above, and incubated overnight at 37 °C under static conditions allowing for biofilm development. Following incubation, plates were washed with 1X phosphate buffered saline (PBS; pH 7.4) and stained with 0.1% Crystal Violet (Sigma-Aldrich, St. Louis, MI, USA). Excess crystal violet was removed by washing with PBS and the amount of attached biofilm was determined by measuring the optical density of crystal violet solubilized in ethanol at 570 nm. Increases in biofilm was reported as a percentage of biofilm biomass, as determined by measurement of the absorbance of crystal violet stain in the treatment groups to that of the non-treated control groups. Experimental values, prior to calculation of % increase in biofilm biomass, were normalized to a media only control containing corresponding increasing amounts of exogenous iron sources only.

### 4.4. Activity of Gallium Compounds on Planktonic Bacteria

Activity of gallium compounds against planktonic bacteria was performed using broth microdilution assays as previously described with minor modifications [32]. Briefly, 10^5^ CFU of bacteria were dispensed into individual wells of the 96-well plate containing increasing concentrations (0–512 µM) of Ga(NO_3_)_3_, Ga-MPIX, or Ga-PPIX diluted in iron depleted medias, 0.3 g/L MHB II or RPMI 1640. Microtiter plates were incubated at 37 °C for up to 24 h. Following incubation, bacterial viability was determined by plating serial dilutions onto Mueller Hinton agar. In brief, bacteria were serially diluted (1:10) in sterile saline. Ten microliters of the diluted bacteria were then plated on to MHB plates for quantification [53]. Concentrations of gallium compounds reducing viable bacteria to 50% and 90% compared to the untreated control were reported as the inhibitory concentration 50 and 90 (IC_50_ and IC_90_, respectively). Experimental assays were performed in duplicate for each strain tested in both media.

### 4.5. Assessment of the Antibiofilm Activity of Gallium Compounds

The activity of gallium compounds on *A. baumannii* biofilms were evaluated using the MBEC P & G plates (Innovotech, AB, Canada) as previously described [37]. Bacteria and biofilms were grown as described above for the 96-well plate assays with the exception that MBEC plates, containing a lid with pegs for bacterial attachment and biofilm formation, were used. Briefly, bacteria (~10^6^ CFU) were added to individual wells of the MBEC plate and incubated overnight at 37 °C for 24 h on an orbital shaker (VWR, Radnor, PA, USA). To assess the activity of gallium compounds on bacteria within established biofilms, the lids containing the pegs with preformed biofilms, were removed, washed in PBS, and placed into a challenge plate containing 0–512 µM Ga(NO_3_)_3_, Ga-MPIX, or Ga-PPIX diluted into either 0.3 g/L MHB II or RPMI. The plates were then incubated at 37 °C for up to 24 h. Following treatment, pegs were rinsed, placed into a new 96-well plate containing saline, and sonicated for 15 min at 40 kHz (Branson Ultrasonics Corp, Danbury, CT, USA) to remove the attached bacteria. Bacterial viability was determined by plating serial dilutions onto MHB agar plates. Serial dilutions to determine bacterial viability were performed in a similar manner as described above in Section 4.4. Concentrations of the gallium compounds reducing bacterial viability to 50% and 90% compared to the untreated control were reported as the minimum biofilm eradication concentration 50 and 90 (MBEC_50_ and MBEC_90_, respectively). Assays were performed in duplicate for each strain tested in both media.

### 4.6. Cellular Viability Assays

Cellular viability assays were performed to evaluate the cytotoxicity of the gallium compounds as previously described [54], which, in brief, consisted of exposing confluent monolayers of human dermal fibroblasts (PromoCell, Heidelberg, Germany) seeded into 96-well black clear bottom plates, to 0–512 µM Ga(NO_3_)_3_, Ga-MPIX, and Ga-PPIX diluted in Dulbecco Modified Eagle Medium (DMEM), supplemented with 5% fetal bovine serum, 10 U/mL of penicillin, and 10 μg/mL streptomycin, and incubation overnight at 37 °C in 5% CO_2_. Following exposure, cells were washed, resuspended in PBS, and cell viability was measured using Cell Titer Fluor assay (Promega, Madison, WI, USA) as recommended by the manufacturer. Cell viability was reported as a percentage of the non-treated control group, exposed to culture media only.

### 4.7. LDH Assays

Supernatants were collected from the treated cell cultures described above. Cytotoxicity was then determined by measuring the levels of released lactate dehydrogenase (LDH) within supernatants using an LDH cytotoxicity assay kit (Thermo Scientific, Grand Island, NY, USA) according to the manufacturer’s instructions. In brief, extracellular LDH activity was determined indirectly by spectrophotometrically measuring the levels of reduced tetrazolium salt (INT) at 490 nm. Cytotoxicity was then determined, by subtracting the spontaneous LDH release (media only control) from the LDH in the treated samples, divided by the total LDH activity (positive control) and reported as a ratio of toxicity.

### 4.8. Statistical Analysis

Statistical analysis was performed using the Student’s *t*-test and one-way ANOVA for multigroup comparisons followed by a Dunnett’s *post hoc* test for comparisons between test and control groups using Graphpad Prism, version 5. Differences were considered to be statistically significant at *p* values of <0.05.

## 5. Conclusions

In conclusion, we have demonstrated that the gallium porphyrin conjugates in comparison to Ga(NO_3_)_3_ have better antibacterial and antibiofilm activity against genotypically diverse clinical isolates of MDR *A. baumannii*. Limited toxicity of the gallium porphyrin conjugates at the effective antibacterial concentrations indicates their potential use as an unconventional antimicrobial strategy for *A. baumannii* infections. Additional studies should confirm these findings *in vivo* and explore potential delivery devices that could be used for clinical treatment.

## Figures and Tables

**Table 1:** Characteristics of strains used in this study.

Isolate (*A. baumannii*)	Source	Specific Site	Phenotype ^a^	Pulse Field Type (PFT)	Colistin Resistance ^b^
AB 1	Wound	Pelvis	MDR	51	N
AB 2	Wound	Forearm	MDR	50	N
AB 3	Wound	Forearm	MDR	7	N
AB 4	Wound	Lower Extremity	MDR	50	N
AB 5	Wound	Upper Extremity	MDR	51	N
AB 6	Wound	Pelvis	MDR	51	N
AB 7	Wound	Upper Extremity	MDR	44	N
AB 8	Wound	Pelvis	MDR	50	N
AB 9	Wound	Lower Extremity	MDR	50	N
AB 10	Wound	Upper Extremity	MDR	44	N
AB 11	Blood	---	PDR	49	Y (MIC >256 µg/mL)
AB 12	Wound	Lower Extremity	PDR	44	Y (MIC 12 µg/mL)
ATCC 17978	Meningitis	Unknown	Non-MDR	Unknown	N

^a^ A multidrug resistant (MDR) organism was defined as an organism resistant to antimicrobials in 3 or more classes of antimicrobial agents (penicillins/cephalosporins, carbapenems, aminoglycosides, and quinolones) not including tetracyclines or colistin. A pandrug resistant (PDR) organism was considered to be a strain displaying MDR resistance in addition to colistin resistance. ^b^ MICs were determined by the E-test method according to the manufacturer’s guidelines, a ≥4 ug/mL colistin concentration was used as the breakpoint to designate resistant isolates [48].
